# Human milk microbiota and oligosaccharides in colostrum and mature milk: comparison and correlation

**DOI:** 10.3389/fnut.2024.1512700

**Published:** 2024-12-12

**Authors:** Hongda Ge, Wenxiu Zhu, Jing Zhang, Zijing Wang, Huijing Shi, Jie Sun, Ming Shi

**Affiliations:** ^1^Department of Clinical Laboratory, Dalian Women and Children’s Medical Group, Dalian, China; ^2^Centre for Reproductive and Genetic Medicine, Dalian Women and Children’s Medical Group, Dalian, China; ^3^Department of Clinical Laboratory, Central Hospital of Dalian University of Technology, Dalian Municipal Central Hospital, Dalian, China; ^4^Maternity Ward, Dalian Women and Children’s Medical Group, Dalian, China; ^5^Child Health Care Clinic, Dalian Women and Children’s Medical Group, Dalian, China

**Keywords:** breast milk, human milk oligosaccharides, human milk microbiota, colostrum, mature milk

## Abstract

**Background:**

The interaction between the human breast milk microbiota and human milk oligosaccharides (HMOs) plays a crucial role in the healthy growth and development of infants. We aimed to clarify the link between the breast milk microbiota and HMOs at two stages of lactation.

**Methods:**

The microbiota and HMOs of 20 colostrum samples (C group, 1–5 days postpartum) and 20 mature milk samples (S group, 42 days postpartum) collected from postpartum mothers were analyzed using 16S rRNA gene high-throughput sequencing and high-performance liquid chromatography–tandem mass spectrometry.

**Result:**

The total average HMO content was significantly higher in the C group than in the S group (6.76 ± 1.40 g/L vs. 10.27 ± 2.00 g/L, *p* < 0.05). Among the HMOs, the average values of 2′-fucosyllactose (2′-FL, 1.64 ± 1.54 g/L vs. 3.03 ± 1.79 g/L), 3′-sialyllactose (3′-SL, 0.10 ± 0.02 g/L vs. 0.21 ± 0.06), 6′-SL (0.22 ± 0.09 g/L vs. 0.33 ± 0.11 g/L), and lacto-N-triaose 2 (LNT2, 0.03 ± 0.01 g/L vs. 0.16 ± 0.08 g/L) were significantly lower in the S group than in the C group (*p* < 0.05), while that of 3′-FL was significantly higher in the S group than in the C group (1.35 ± 1.00 g/L vs. 0.41 ± 0.43 g/L, *p* < 0.05). The diversity and structure of the microbiota in the S and C groups were also significantly different (*p* < 0.05). Comparative analysis of the microbial communities revealed that Proteobacteria and Firmicutes were the most abundant phyla, in both groups, with the keystone species (*Serratia*, *Streptococcus* and *Staphylococcus*) of breast milk closely interacting with HMOs, including 3′-SL, 6′-SL, and LNT2. In PICRUSt2 functional prediction analysis, the S group exhibited significant reduction in the expression of genes involved in several infectious disease pathways.

**Discussion:**

Our findings support the recognition of human milk as a synbiotic comprising beneficial bacteria and prebiotic HMOs.

## 1 Introduction

Breast milk is the most natural and complete food for infant growth (1). In addition to being rich in protein, fat, carbohydrates, vitamins, and water, breast milk also contains immunoglobulins, human milk oligosaccharides (HMOs), cytokines, and hormones. As a group of polysaccharide substances that are unique to humans, with diverse functions and structures, HMOs are characteristically difficult to digest (1). The content of HMOs accounts for about 20% of the total carbohydrate content in breast milk (2). While there are individual changes in HMO over the entire period of breastfeeding (up to 24 months postpartum), the absolute concentration and relative abundance of most of the examined HMOs decreased during lactation. In addition to the previously documented differences in HMO concentrations between secretors and non-secretors, many of the changes in HMO that occur during lactation differ between secretors and non-secretors (3). After ingestion of HMOs, approximately 0.05% of the HMOs enter the bloodstream with the vast majority of HMOs entering the large intestine (4, 5). Owing to their unique carbohydrate structure, they are capable of playing a role in the infant’s resistance to low gastric pH, as well as pancreatic and brush border enzymes (6). Hence, the majority of HMOs are eliminated from the body through feces, with a portion of the HMOs being metabolized—mainly by the infant gut microbiota—to produce various beneficial effects (7). HMOs serve as metabolic substrates for the growth of probiotics such as *Bifidobacterium* in the infant’s gut (8, 9), thereby reducing intestinal pH and inhibiting pathogen growth. Important products of the metabolism of HMOs, including acetic acid and butyric acid, can lower the pH value in the intestine and inhibit the growth of pathogens. In addition to affecting bifidobacteria, other bacteria that have also been studied, including *Lactobacillus acidophilus* and *Eubacterium hallii*, exhibit physiological effects that are all related to HMOs (10). In addition to having nutritional effects on the gut microbiota of infants, there is increasing evidence that HMOs can interact with pathogens to reduce the occurrence of infections (2). The structure of HMOs, which are similar to the surface glycoconjugates on which microorganisms attach to human intestinal cells, can act as bait receptors for binding to pathogens, reducing the adhesion of pathogenic microorganisms to the intestinal wall (11).

Importantly, breast milk also has a microbial ecosystem. Breast milk has traditionally been considered to be a sterile liquid (12). However, using culture medium screening methods, researchers have confirmed the presence of *Staphylococcus* and *Streptococcus* in breast milk collected under sterile conditions. Studies using microbial identification techniques such as metagenomic sequencing and 16S rRNA gene amplification sequencing have shown that breast milk also contains various other bacteria, including *Lactobacillus* and *Bifidobacterium* (13). A systematic review of the bacterial bank in breast milk showed the presence of approximately 820 types of bacteria, mainly belonging to the Gram-positive Firmicutes and Gram-negative Proteobacteria phyla (14). Current studies have shown that the breast milk microbiota is a decisive factor in the development of the gut microbiota and plays a pioneering role in the healthy development of infants (15). From the initial contact of the infant’s gut with the microbiota transferred from breast milk, the microorganisms begin to colonize and grow in the intestine. This is a gradual process that plays a crucial role in the maturation of the intestinal lymphoid tissue and the growth and development of intestinal epithelial cells for infants (16). The microbiota in breast milk mainly reduces the risk of infant infection and disease through competition and inhibition among the microbiota (8). Importantly, maternal diet, age, and different periods of lactation affect the nutrients, bioactive compounds, immune factors, and microbiota contained in breast milk, which further affect the structural changes in the gut microbiota for infants (17, 18).

Human milk oligosaccharides and the breast milk microbiota play important roles in the establishment and development of the infant gut microbiota. The relationship between the changes in HMO concentration that occur during lactation and the microbiota of breast milk is not well-studied. The purpose of this study were to: (1) investigate the differences in the microbial community and the content and types of HMOs in breast milk between colostrum samples and mature milk samples at 42 days postpartum; and (2) analyze correlations between HMOs and the breast milk microbiota. Our findings provide specific experimental evidence for the importance of breastfeeding, which is of great significance for the development of infant formula foods that are closer to breast milk.

## 2 Experimental methods

### 2.1 Participants

In this study, 40 healthy postpartum mothers were recruited at Dalian Women and Children’s Medical Center (Group) for the collection of breast milk samples. Twenty human colostrum samples (C group) were donated within 1–5 days postpartum by 20 mothers, and 20 mature milk samples (S group) were donated by the remaining 20 mothers at 42 days postpartum (19). We also collected maternal demographic and clinical data including age, height, weight, body mass index, number of deliveries, and delivery method (Supplementary Table S1).

Inclusion criteria were as follows: (1) no underlying metabolic disorders; (2) no gastrointestinal diseases, immunological disorders, infectious diseases, or organic diseases. (3) Received no antibiotics within 1 month before breast milk sampling. The study was conducted following the principles of the Declaration of Helsinki, and reviewed by the Medical Ethics Committee of Dalian Women and Children’s Medical Center (approval number: 2022017). Written informed consent was obtained from the mother before the participation in the study.

### 2.2 Breast milk samples collection

Before collecting breast milk samples, the mothers were required to clean their hands with soap and use disinfectant wipes or 75% alcohol to sanitize the nipple and areola surfaces. They were also instructed to discard the first drop of breast milk to prevent contamination. Breast milk samples (more than 5 mL) were collected, placed in a pre-prepared sterile container, sealed, and stored in a −80°C freezer until analysis (20).

### 2.3 High-performance liquid chromatography (HPLC)-tandem mass spectrometry (MS/MS) of HMOs in breast milk

Breast milk samples were blended to mix and brought to room temperature. A 1-mL aliquot of each sample was transferred to a clean, 2-mL centrifuge tube and spun at 4°C under 12,000 rpm for 10 min. After removing the upper oil layer, 1 mL of anhydrous ethanol was added, and the mixture was allowed to stand for 4 min to precipitate protein. After centrifugation at 12,000 rpm for 5 min, the 200-μL supernatant was loaded onto an HPLC-MS/MS machine (Quantum Hi-Tech [Guangdong] Biological Co., Ltd.) for quantitative analysis of each component, alongside the following 16 HMO standards (20): 2′-fucosyllactose (2′-FL), 3′-fucosyllactose (3′-FL), lacto-*N*-tetraose (LNT), lacto-*N*-neotetraose (LNnT), 3′-sialyllactose (3′-SL), 6′-sialyllactose (6′-SL), lactose-*N*-difucohexose I (LNDFH I), lactose-*N*-difucotetraose (LDFT), lactoyl-*N*-fucopentaose I (LNFP I), disialylated lactose-*N*-tetrasaccharide (DSLNT), sialyl-lactose-*N*-tetrasaccharide (LSTc), bisfucosyllactose-*N*-hexasaccharide (DFLNH), lactose-*N*-difucohexose II (LNDFH II), and lactoyl-*N*-fucopentaose II (LNFP II). HMOs are mainly divided into three structural types: neutral fucosylated HMOs, neutral non-fucosylated HMOs, and acidic sialylated HMOs. Neutral fucosylated HMOs mainly include 2′-FL and 3′-FL; neutral non-fucosylated HMOs include LNT and LNnT; and acidic sialylated HMOs include 3′-SL and 6′-SL (21, 22).

### 2.4 Nucleic acid extraction and Illumina sequencing of human breast milk microbiota DNA

Total DNA was isolated from breast milk samples (1.5–2.0 mL) using the E.Z.N.A.^®^ Stool DNA Kit (Epicentre), in accordance with the manufacturer’s instructions. The V3–V4 hypervariable variable regions of genomic DNA samples were amplified by polymerase chain reaction (PCR) using primers 338F (5′-ACTCCTACGGGAGGCAGCA-3′) and 806R (5′-GGACTACHVGGGTWTCTAAT-3′). PCR products of breast milk microbiota composition were determined by V3–V4 variable regions of the 16S rRNA gene high-throughput sequencing by Shanghai Maggi Biomedical Technology Co., Ltd., using an Illumina MiSeq PE300 following Illumina protocols. The 16S rRNA gene sequences were defined as one operational taxonomic unit (OTU) based on 97% similarity. The abundance-based coverage estimator (Ace) index, observed species (Sobs) index, and Shannon diversity index were used to reflect the alpha diversity of samples. A hierarchical clustering tree at the OTU level, principal component analysis (PCoA), and non-metric multidimensional scaling analysis (NMDS) were used to analyze beta diversity. Linear discriminant analysis (LDA) was used to screen for dominant microbial communities (23). Random Forest model analysis was performed on the Maggi Platform under default settings through the utilization of an out-of-bag error within the R environment (version 3.3.1). The data were standardized at the genus level by the Z-score method, and the area under the curve (AUC) was verified under the condition that the number of with 500 decision trees (24). Phylogenetic Investigation of Communities by Reconstruction of Unobserved States (PICRUSt) was applied to predict functional profiles of the gut microbiota resulting from reference-based OTU picking against the Greengenes database. The predicted genes were then summarized using the Kyoto Encyclopedia of Genes and Genomes (KEGG) pathway categorization. The difference-rich OTUs were employed to calculate Pearson’s correlation coefficients, which were used to conduct network analysis. Gephi was used for topology analysis and visualization purposes (23).

### 2.5 Statistical analysis

The experimental data and statistical charts in this study were analyzed and processed using SPSS 22 software (IBM, United States) and Graph Pad Prism Version 8 (Graph Pad Software Inc., United States). Statistical analysis of KEGG pathway data was performed with STAMP v2.1.3 using Welsh’s *t*-test (*p* < 0.05). The experimental data were expressed as mean ± standard deviation (SD), with a *p-*value <0.05 indicating statistical significance; *p < 0.05, **p < 0.01 and ***p < 0.001 vs. the M group.

## 3 Results

### 3.1 Basic clinical information

Analysis of the basic clinical data of the study participants showed no significant differences in age or body mass index (BMI) between the C and S groups (Table 1 and Supplementary Table S1).

**Table 1:** Clinical information.

Parameters		The colostrum group (*n* = 20)	Mature breast milk (42 days postpartum) (*n* = 20)
Age		31.61 ± 5.46	31.2 ± 4.14
BMI index		27.83 ± 3.80	23.35 ± 4.18
Number of deliveries	1 birth	65%	85%
	2 fetuses	35%	15%
Mode of deliveries	Vaginal birth	35%	45%
	Cesarean section	65%	55%
Whether to use antibiotics		no	no

Age and BMI are expressed as mean ± standard deviation. Body Mass Index (BMI) = weight (Kg)/height ^ 2 (m).

### 3.2 Analysis of HMOs in breast milk

**Figure 1:**
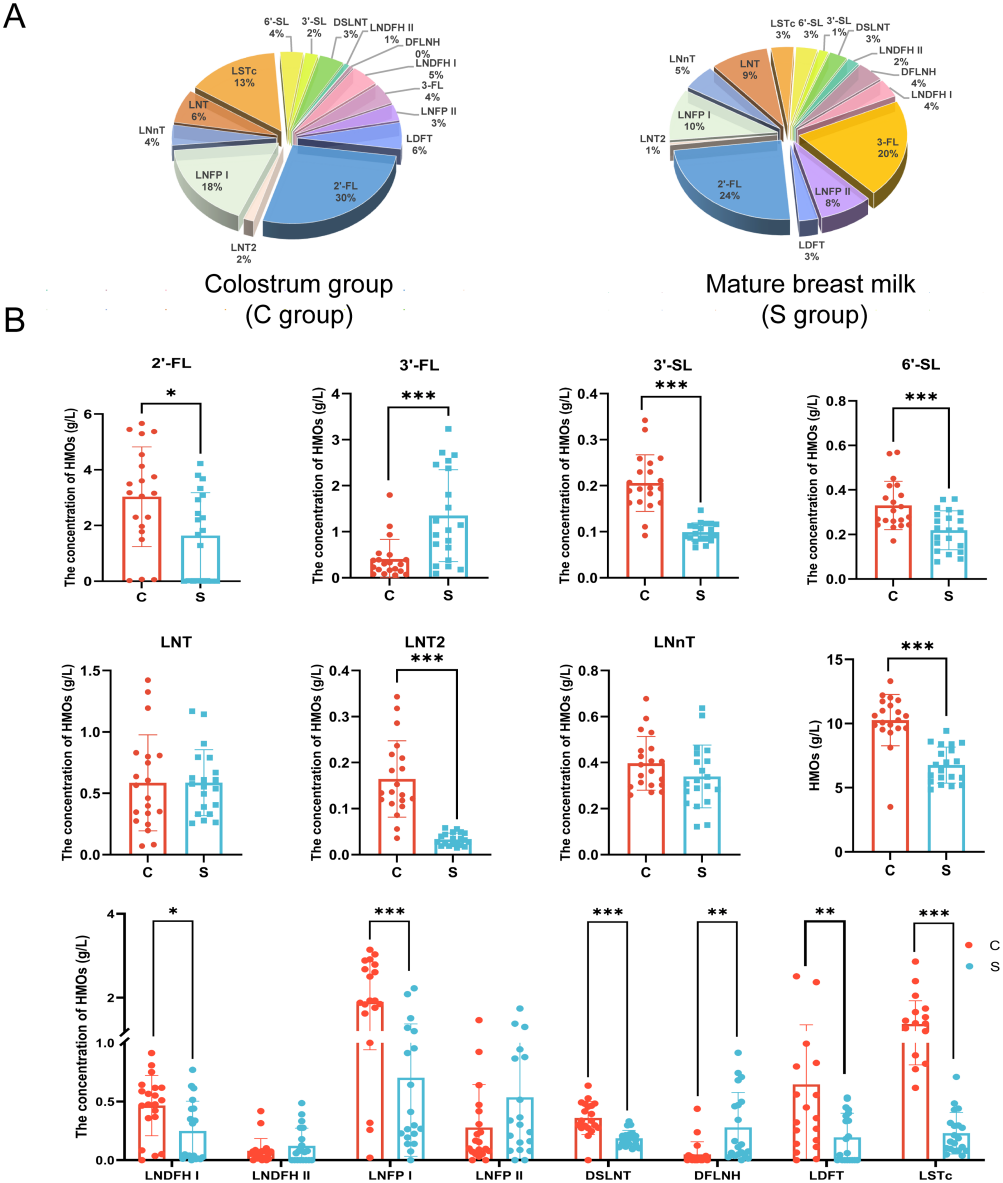
The proportion **(A)** and composition **(B)** of oligosaccharides in breast milk. C: the colostrum group (*n* = 20); S: the mature milk (42 days postpartum) group (*n* = 20). ^*^p < 0.05, ^**^p < 0.01, ^***^p < 0.001 compared with the C group.

As shown in Figure 1A, there were differences in the concentrations of individual HMOs in breast milk between the C and S groups. In both study groups, the top five most abundant individual HMOs identified were all found to belong to the fucosylated group: 2′-FL (30%), LNFPI (18%), LSTc (13%), LNT (6%), and LDFT (6%) in the C group; and 2′-FL (24%), 3′-FL (20%), LNFP I (10%), LNT (9%), and LNFP II (8%) in the M group. The average total HMO content in breast milk was significantly lower in the S group than in the C group (6.76 ± 1.40 g/L vs. 10.27 ± 2.00 g/L, *p* < 0.05; Figure 1B). Among the neutral fucosylated HMOs, the average concentration of 2′-FL was significantly lower in the S group than in the C group (1.64 ± 1.54 g/L vs. 3.03 ± 1.79 g/L, *p* < 0.05), whereas the average concentration of 3′-FL was significantly higher in the S group than in the C group (1.35 ± 1.00 g/L vs. 0.41 ± 0.43 g/L, *p* < 0.05; Figure 1B). Among the neutral non-fucosylated HMOs, the average concentration of LNT2 was significantly lower in the S group than in the C group (0.03 ± 0.01 g/L vs. 0.16 ± 0.08 g/L, *p* < 0.05), but there was no significant differences in those of LNT (0.59 ± 0.27 g/L vs. 0.59 ± 0.39 g/L) and LNnT (0.34 ± 0.14 g/L vs. 0.40 ± 0.12 g/L) between the two groups (*p* > 0.05). The average concentrations of the acidic sialylated HMOs 3′-SL (0.10 ± 0.02 g/L vs. 0.21 ± 0.06 g/L) and 6′-SL (0.22 ± 0.09 g/L vs. 0.33 ± 0.11 g/L) were significantly lower in the S group than in the C group (*p* < 0.05). The average concentrations of LNDFH I (0.25 ± 0.25 g/L vs. 0.47 ± 0.26 g/L), LDFT (0.19 ± 0.21 g/L vs. 0.65 ± 0.71 g/L), LNFP I (0.70 ± 0.67 g/L vs. 1.90 ± 0.96 g/L), DSLNT (0.19 ± 0.07 g/L vs. 0.36 ± 0.14 g/L), and LSTc (0.23 ± 0.18 g/L vs. 1.37 ± 0.56 g/L) were significantly lower in the S group than in the C group (*p* < 0.05). The average concentration of DFLNH was significantly higher in the S group than in the C group (0.28 ± 0.30 g/L vs. 0.05 ± 0.11 g/L, *p* < 0.05). There were no significant differences in the average concentrations of LNDFH II (0.12 ± 0.15 g/L vs. 0.08 ± 0.10 g/L) and LNFP II (0.54 ± 0.52 g/L vs. 0.28 ± 0.37 g/L) between the C and S groups, respectively (*p* > 0.05).

### 3.3 Quality assessment of human breast milk microbiota DNA samples and 16S rRNA gene amplification and sequencing

**Figure 2:**
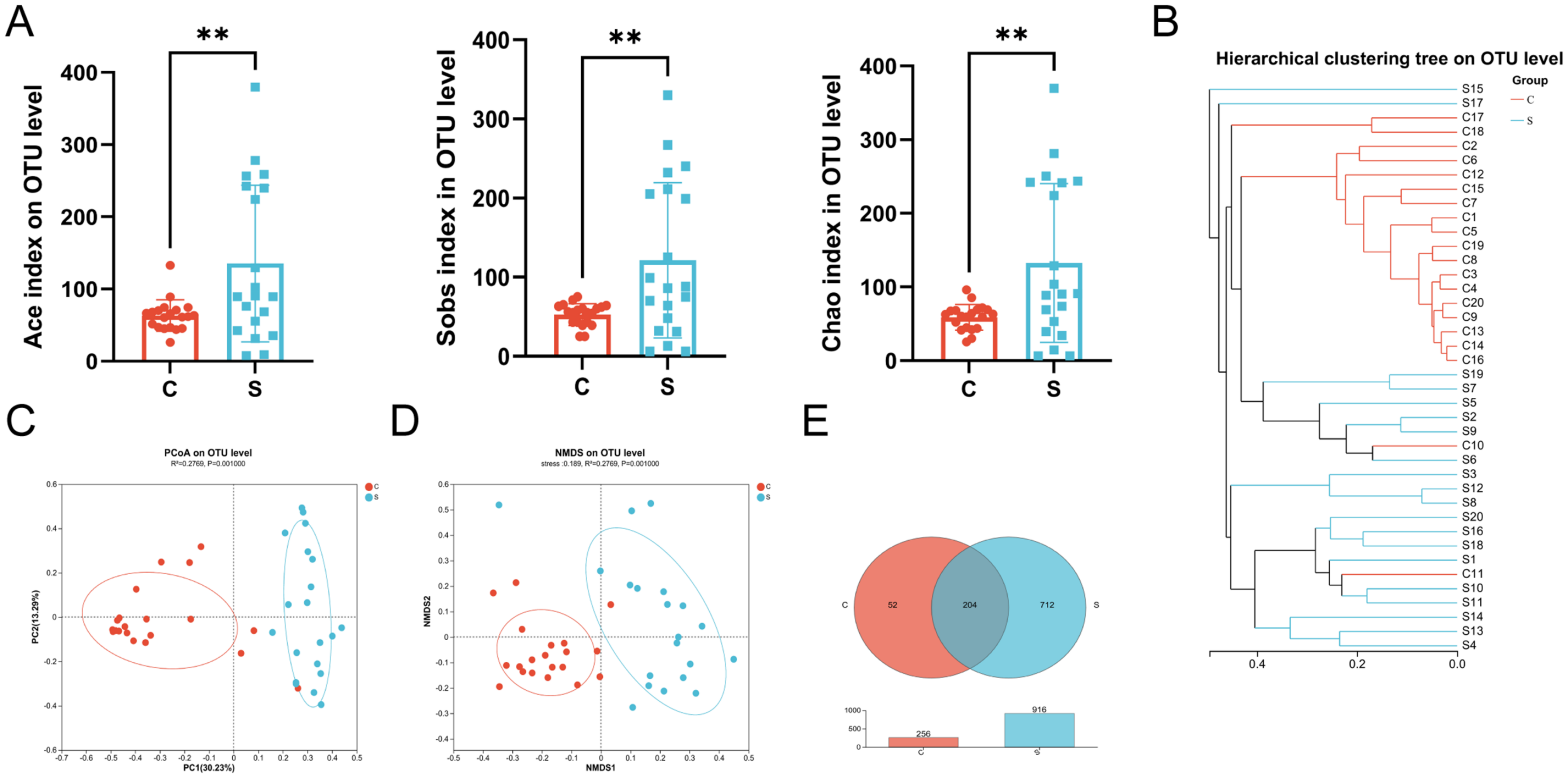
The microbial diversity and composition of the breast milk microbiota in the C and S group. **(A)** Alpha diversity (Ace and Sobs and Chao index) on the OTU level. **(B)** Hierarchical clustering tree on the OTU level. **(C)** Principal component analysis (PCoA) with cluster. **(D)** Non-metric multidimensional scaling analysis (NMDS) with cluster. **(E)** Venn diagram. C: the colostrum group (*n* = 20); S: the mature milk (42 days postpartum) group (*n* = 20). ^*^p < 0.05, ^**^p < 0.01, ^***^p < 0.001 compared with the C group.

Comparison of the results of 16S rRNA gene high-throughput sequencing analysis of the human breast milk microbiota in the C and S groups (Supplementary Table S2) showed stabilization of the refraction curve of the Sobs and Shannon indexes (Supplementary Figure S1), indicating sufficient depth of sequencing data for each group of samples. Similarly, the Good’s coverage index of the breast milk microbiota reached above 0.99 (Supplementary Table S2). The Ace, Sobs, and Chao indexes of the S group showed significantly higher alpha-diversity compared with those of the C group (*p* < 0.01; Figure 2A). There was no difference between the Shannon index and Simpson index values (Supplementary Table S3).

The beta-diversity of the breast milk microbiota was demonstrated by the formation of two distinct clusters between the C and S groups, as illustrated by hierarchical clustering, PCoA (*R*^2^ = 0.2769, *p* = 0.001) and NMDS (Stress = 0.189, *R*^2^ = 0.2769, *p* = 0.001) analysis at the OTU level (Figures 2B–D). Meanwhile, there were 204 shared OTUs between the groups. The S group had 712 unique OTUs, which far exceeded the 53 OTUs in the C group, indicating that the number of OTUs in the S group increased with time (*p* < 0.01; Figure 2E).

### 3.4 Analysis of the composition of microbial communities in colostrum and mature milk

**Figure 3:**
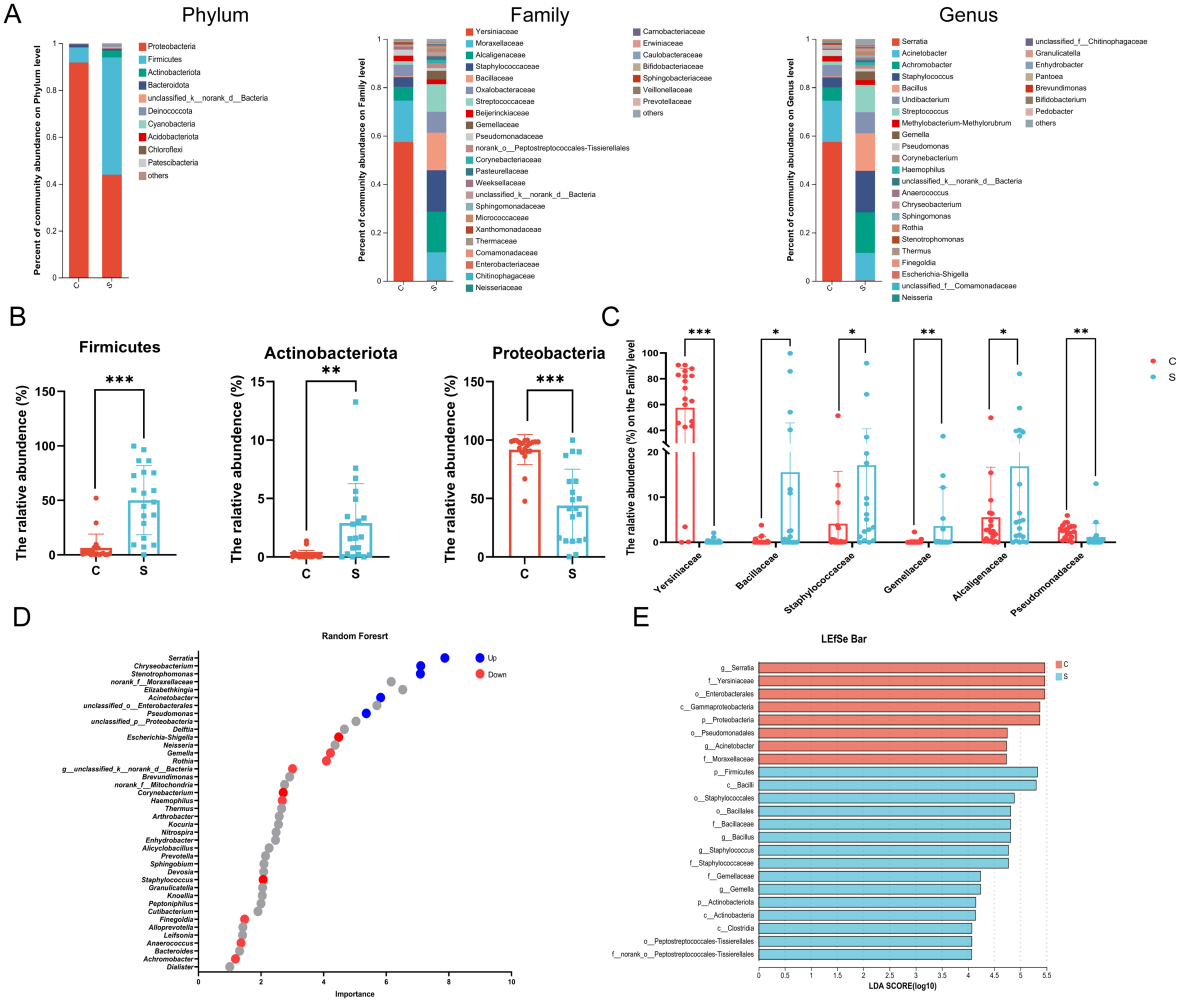
Analysis of the structure and communities of the breast milk microbiota in the N and M group. **(A)** Microbial distributions of different groups at the phylum, Family and Genus level. **(B)** The relative abundance of Firmicutes, Actinobacteria and Proteobacteria at the Phylum level. **(C)** The relative abundance of *Alcaligenaceae*, *Bacillaceae*, *Gemellaceae*, *Pseudomonadaceae*, *Staphylococcaceae*, and *Yersiniaceae* at the Family level. **(D)** Random forest at the genus level. Up (red) represented the S group raised; down (blue) represented the S group decreased (*p* < 0.05). **(E)** Linear discriminant analysis (LDA) distribution by analyzing Linear discriminate analysis effect size (LEfSe). The threshold of the LDA score was 4.0. C: the colostrum group (*n* = 20); S: the mature milk (42 days postpartum) group (*n* = 20). ^*^p < 0.05, ^**^p < 0.01, ^***^p < 0.001 compared with the C group.

At the phylum level, the microbial community of the C group was mainly composed of Proteobacteria, Firmicutes, and Bacteroidota, while that of the S group was mainly composed of Proteobacteria, Firmicutes, and Actinobacteria (Supplementary Figure S2A). The relative abundance of Proteobacteria in the breast milk was significantly higher in the C group than in the S group (*p* < 0.05; Figures 3A,B). The relative abundance of Firmicutes and Actinobacteria in the breast milk is significantly higher in the S group than in the C group (*p* < 0.05; Figures 3A,B). At the Family level, the relative abundance of *Alcaligenaceae*, *Staphylococcaceae*, *Bacillaceae*, *Streptococcaceae* and *Gemellaceae* in the S group was significantly higher than that in the C group (*p* < 0.05, Figure 3C). The abundance of *Yersiniaceae* and *Pseudomonas* was significantly higher in the C group than in the S group (*p* < 0.05, Figure 3C). Combining Random Forest analysis and the Wilcoxon rank-sum test at the genus level, the abundance of *Serratia*, *Acinetobacter*, and *Pseudomonas* were significantly higher in the C group than in the S group (*p* < 0.05; Figure 3D and Supplementary Figures S2B,D). The abundances of the genera *Achromobacter*, *Staphylococcus*, *Bacillus*, *Streptococcus*, *Escherichia-Shigella*, and *Gemella* genera were significantly higher in the S group than that in the C group (*p* < 0.05).

Linear discriminant analysis effect size (LefSe) analysis was employed to identify biomarker bacterial genera among two groups using a LDA score = 4. The significant biomarker bacteria in the C group were identified as p_Proteobacteria, *o_Enterobacteriales*, *o_Pseudomonas*, *f_Yersiniaceae*, *f_Moraxellaceae*, *g_Serratia* and *g_Acinetobacter*. The relative abundance of p_Firmicutes, p_Actinobacteriota, *f_Bacillaceae*, *f_Staphylococcaceae*, *f_Gemellaceae*, *f_Histostreptococcales*, *g_Bacillus*, *g_Staphylococcus*, and *g_Gemella* were enriched in the S group (Figure 3E).

### 3.5 Different potential functions and identification of co-abundance networks of colostrum and mature milk at the OTU level

**Figure 4:**
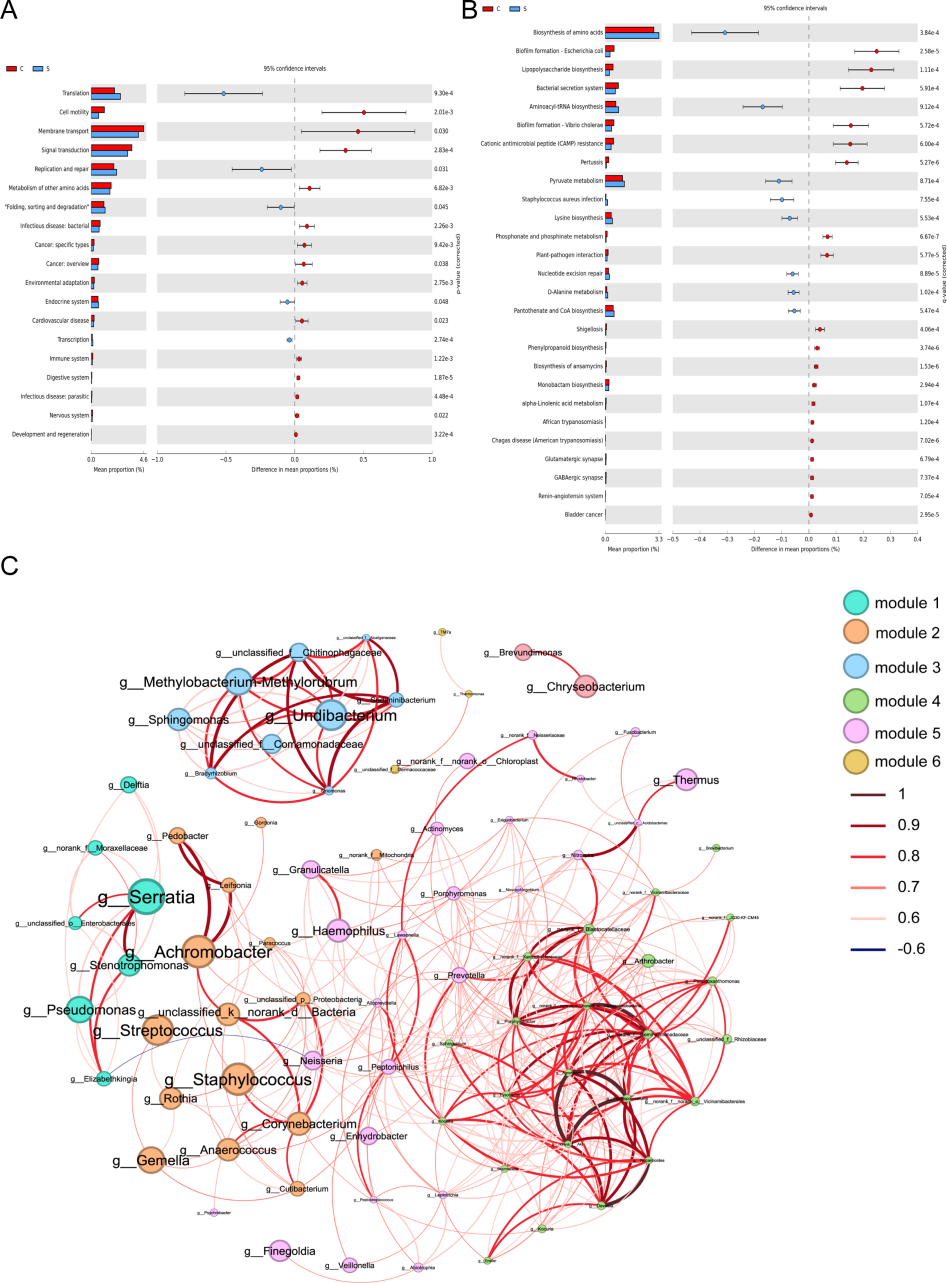
Functional prediction and co-abundance networks at the genus level between the C group and the S group. **(A)** STAMP analysis for the inferred metabolic pathway in level 2. **(B)** STAMP analysis for the inferred metabolic pathway in level 3. **(C)** The co-abundance networks. C: the colostrum group (*n* = 20); S: the mature milk (42 days postpartum) group (*n* = 20). ^*^p < 0.05, ^**^p < 0.01, ^***^p < 0.001 compared with the C group.

The impact of the stage of breast milk on various KEGG pathways was assessed via PICRUST2 analysis of the 16s rRNA amplicon sequencing data using PICRUST2. This revealed significant reductions in the expression of genes related to the following KEGG level 2 functional pathways in mature milk compared with those in colostrum: infectious disease (bacteria, parasitic), cancer (specific types, overview), cardiovascular disease, and nervous system (*p* < 0.05; Figure 4A). The analysis of level 3 KEGG pathways revealed a significant increase in biosynthesis of amino acids, aminoacyl-tRNA biosynthesis, pyruvate metabolism, *Staphylococcus aureus* infection, lysine biosynthesis, nucleotide excision repair, d-alanine metabolism and pantothenate and CoA biosynthesis in the S group compared with those in the C group (*p* < 0.05; Figure 4B). In contrast, several infection-related pathways were reduced in the S group, including biofilm formation-*Escherichia coli*, lipopolysaccharide biosynthesis, bacterial secretion system, biofilm formation-*Vibrio cholera*, pertussis, phosphonate and phosphinate metabolism, plant–pathogen interaction, Shigellosis, phenylpropanoid biosynthesis, biosynthesis of ansamycins, monobactam biosynthesis, alpha-linolenic acid metabolism, African trypanosomiasis, chagas disease (American trypanosomiasis), glutamatergic synapse, and GABAergic synapse (*p* < 0.05; Figure 4B).

Functional prediction analysis fails to capture the complexity of bacterial interactions in microbial communities. Therefore, we sought to explore the inherent patterns of Co-occurrence or Co-exclusion of specific microbial communities driven by spatial and temporal changes and environmental processes at the genus level. This network analysis revealed 84 nodes and 377 edges. Among these, *Serratia*, *Streptococcus* and *Staphylococcus* as the keystone species exhibiting a close-positive association with other genera including *Achromobacter*, *Leifsonia*, *Neisseria*, and *Delfia* (Figure 4C and Supplementary Table S4).

### 3.6 Correlation analysis between HMOs and the breast milk microbiota

In the heatmap based on Spearman correlation analysis at the family level (Supplementary Figure S3), *Lactobacillaceae* was negatively correlated with LNFP II (*p* < 0.05). *Staphylococcaceae* and *Gemellaceae* were negatively correlated with 3′-SL, LNT2, LSTc, and total HMOs (*p* < 0.05). *Yersiniaceae*, *Pseudomonadaceae*, and *Xanthomonadaceae* were positively correlated with 2′-FL, DSLNI, 3′-FL, LNT2, LSTc, LNFP I and HMOs, and negatively correlated with LNFP II, DFLNH, and 3′-FL (*p* < 0.05). *Alcaligenaceae* and *Bacillaceae* was positively correlated with 6’-SL (*p* < 0.05) and DFLNH (*p* < 0.05), respectively.

**Figure 5:**
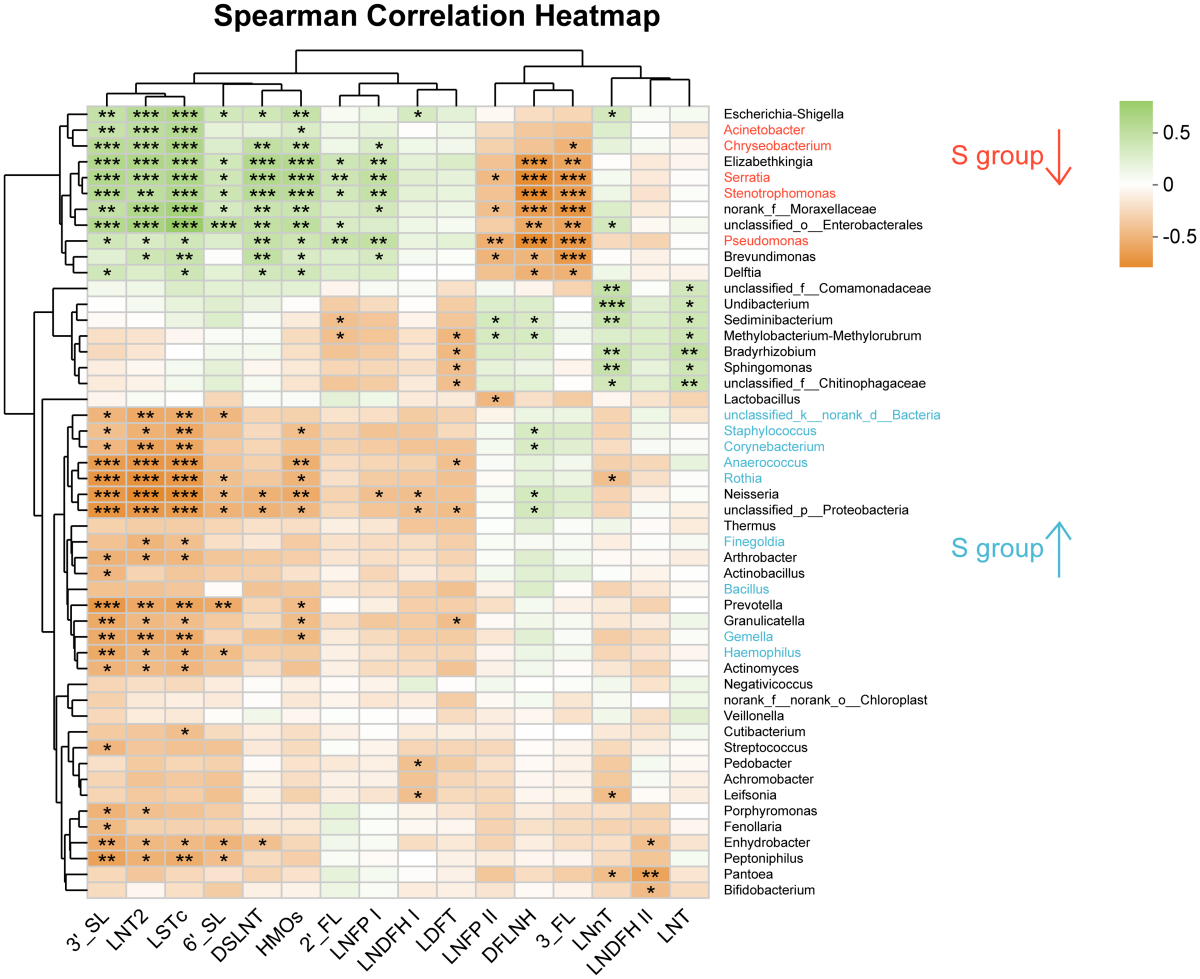
Analysis of the correlation between oligosaccharides and the breast milk microbiota in the C group and the S group at the Genus level. C: the colostrum group (*n* = 20); S: the mature milk (42 days postpartum) group (*n* = 20). ^*^p < 0.05, ^**^p < 0.01, ^***^p < 0.001.

At the genus level, *unclassified_k_norank_d_Bacteria*, *Staphylococcus*, *Corynebacterium*, *Anaerococcus*, *Rothia*, *Gemella*, *Neisseria*, *Acinetobacter* and *Haemophilus* were negatively correlated with 3′-SL, LNT2, and LSTc (*p* < 0.05; Figure 5). *Staphylococcus* was negatively correlated with HMOs (*p* < 0.05), while *Finegoldia* was negatively correlated with LNT2 and LSTc (*p* < 0.05). *Serratia*, *Pseudomonas*, and *Stenotrophomonas* were positively correlated with 3′-SL, LNT2, LSTc, DSLNT, and HMOs (*p* < 0.05), and were negatively correlated with DFLNH and 3′-FL (*p* < 0.05; Figure 5). *Serratia* and *Pseudomonas* were positively correlated with 6′-SL (*p* < 0.05; Figure 5). *Bifidobacterium* was negatively correlated with LNDFH II (*p* < 0.05; Figure 5).

## 4 Discussion

Breast milk is the cornerstone of healthy growth for infants, offering a wide range of benefits. HMOs, which are considered prebiotics (25, 26), are the third most abundant nutrient in breast milk, after lactose and lipids (27). Previous data have shown significant differences in the total HMO concentration in breast milk among individuals. Additionally, previous studies have shown that the concentration of HMOs is highest in colostrum (average 9–22 g/L) and slightly lower in transitional milk (average 8–19 g/L from post-birth days 8 to day 15). As lactation progresses, the concentration of HMOs in mature breast milk gradually decreases, from 6 to 15 g/L in milk collected within 1 month to 4–6 g/L in milk collected within 6 months post birth (2, 7). This is consistent with the results of this study, in which we found a significant difference in the average concentrations of HMOs between colostrum (9.7 g/L) and mature milk (6.8 g/L). However, a study conducted by Kunz et al. (28) found no difference in total HMO concentrations between colostrum, transition milk, and mature milk. This discrepancy may be attributable to differences in sample preparation, detection methods, and data analysis. The current detection methods for HMOs are not uniform. Elwakiel et al. (29) used the capillary laser-induced fluorescence method, Ma et al. (30) employed the HPLC-MRM-MS method, and Huang et al. (31) used the ultra-HPLC-fluorometric detection method.

The most abundant HMO is 2′-FL, accounting for approximately 20–40% of the total HMO content in colostrum (30). This is consistent with the results of our study, in which 2′-FL was found to account for 27% of the total HMO content. As lactation progresses, the concentration of HMOs in breast milk continuously changes with most HMOs decreasing over time. Six HMOs (2′-FL, LNnT, difucosyllactose, lacto-*N*-tetraose, 3′- and 6′-SL) have been observed to enrich butyrate-producing bacteria and *Bifidobacteriaceae in vitro* simulating microbial ecology and the gut epithelium (32). One exception is 3′-FL, which increases along the length of the intestine and stimulates the production of mucin and antimicrobial peptides in intestinal goblet cells, benefiting the immunity of the infant’s gut (33). Consistent with the results of this study, previous studies reported a negative correlation between 2′-FL and 3′-FL (2, 34), mainly attributable to the two HMOs containing similar molecular structures and sharing guanosine 5′-diphosphate-1-amylose (GDP) as a substrate (35). LNT2 can be obtained through the acid hydrolysis of LNnT, which can quickly increase the abundance of beneficial bacteria and reduce the abundance of pathogenic bacteria, affecting the healthy balance of the human colon microbiota (36). In a study by Kong et al. (36), 90.1% of LNT2 was utilized by the infant fecal microbiome *in vitro*, effectively increasing the production of acetic acid, succinic acid, lactic acid, and butyric acid, and significantly increasing the number of *Bifidobacterium* cells (37). Although the trend of its concentration change was barely mentioned by the authors, the study showed that LNT2 concentration increased with the prolongation of lactation time. Conversely, the concentration of DFLNH reportedly gradually decreases with lactation (38). This is inconsistent with the results of our study, in which the concentration of DFLNH was significantly higher in mature milk than in colostrum, and may be attributable to factors including race and lifestyle habits. Furthermore, we found that the concentrations of two acidic sialylated HMOs (3′-SL and 6′-SL) decreased with the duration of lactation. The 6′-SL and 2′-FL have previously been shown to protect against necrotizing enterocolitis (severity) by reducing inflammation through inhibition of the Toll-like receptor 4 activation pathway (39). Additionally, in the newborn piglet model, 3′-SL and 6′-SL administration were shown to up-regulate the level of glia-derived neurotrophic factor (GDNF) and the phosphorylation of CAMP response element binding protein (CREB) on the extracellular domain of neural cell adhesion molecule (NCAM) in the ileum, thereby controlling neuronal function to promote intestinal maturation (40, 41).

Recent studies have demonstrated that, while breast milk contributes to approximately 25% of an infant’s gut microbiota (41), its impact on short-and long-term infant health remains uncertain (42). Nonetheless, limited research has established a definitive correlation between the breast milk microbiota and HMOs. This prompted our interest in investigating the regulation of the human milk microbiota and its association with HMOs during lactation. Over time, the diversity and richness of the breast milk microbiota continue to decrease, mainly due to changes in the nutritional composition of breast milk (43, 44). The major bacterial phyla identified in this study included Firmicutes and Proteobacteria consistent with published results (45–47). The breast milk microbiota is the main source of early colonization in the gut of infants (48–50). Here, we found that *Achromobacter*, *Staphylococcaceae*, *Bacillaceae*, *Streptococcaceae*, *Gemellaceae*, *Yersiniaceae*, and *Pseudomonas* accounted for most of the microbiota in breast milk at the family level consistent with those at the genus level. During the course of lactation, we observed increases in the relative abundance of *Achromobacter*, *Acinetobacter*, *Staphylococcus*, *Bacillus*, and *Streptococcus* within the human milk microbiota. The gut microbiota of neonates was positively correlated with most of the microorganisms in the human milk microbiota, which included *unclassified_Enterobacteriaceae*, *Escherichia-Shigella*, *Streptococcus*, and *Bifidobacterium*, suggesting that human milk microbiota may promote neonatal intestinal colonization (51). In contrast, a previous study found the dominant genera, including *Acinetobacter*, *Streptococcus*, *Enterobacteriaceae*, *Serratia*, *Chryseobacterium*, *Prevotella*, and *Corynebacterium*, did not change significantly between the different lactation periods (52). Our finding showed that significant negative correlation between *Acinetobacter* and 3′-SL, LNT2, and LSTc. Studies have confirmed the antibacterial and anti-biofilm activities of HMOs against various bacteria *in vivo*, including *S. agalactiae*, *Staphylococcus aureus*, and *Pseudomonas aeruginosa* (53). We also found a negative correlation between HMOs and *Staphylococcus*, consistent with the findings of Moossavi et al. (54). In contrast, Williams et al. found a positive correlation between the relative abundance of *S. aureus* and HMO content of healthy breast milk samples (55).

*Serratia* and *Escherichia-Shigella* have been shown to be abundant in human breast milk and infant feces (56). The gradual decrease in *Serratia* in break milk during lactation may reduce its pathogenic risk to infants, especially for premature infants, who face a higher risk of infection. However, a study of prodigiosin produced by *Serratia* sp. C6LB and isolated from milk yield inconsistent findings, exhibiting significant biological activities including antibacterial properties, anti-biofilm effects, and cytotoxicity (57). By combining the results from Random Forest, LefSe, and network analyses, we found that *Serratia*, *Staphylococcus*, and *Streptococcus* were the core genera of the breast milk microbiota, consistent with the results of Lackey et al. (17, 58). Furthermore, at the genus level, the top 50 bacteria, which included *Serratia*, *Staphylococcus*, and *Streptococcus*, were almost associated with 3′-SL, LNT2, LSTc, 6′-SL, and DSLNT. Additionally, KEGG analysis identified several infectious disease pathways with significantly decreased activity in mature milk, suggesting that the microbial community in breast milk may possess anti-infective properties, which could be closely related to the abundant HMOs in breast milk. HMOs and the human milk microbiota complement each other to impact the gut microbiota of infants, benefiting specific bacterial genera (55). The HMOs can directly mediate *Staphylococcus*, *Streptococcus*, *Lactobacillus*, and *Enterococcus* in the infant intestine to regulate maternal mammary epithelial cell responses and local immune responses (59). Neonatal breast milk HMOs such as 3′-SL and 6′-SL have the higher correlation with the gut microbiota compared with those of 5-month-old breast milk (60). We also observed a negative correlation between *Lactobacillaceae* or *Bifidobacterium*, which had a low relative abundance (about 2–3%), and the mother’s own HMOs (LNDFH II or LNFP II) in both colostrum and mature milk groups. It was previously shown that *Lactobacillus* levels were negatively correlated with LNFP II and LNDFH II, which is consistent with the results presented here (61). LNDFH II can be enzymatically produced from LNT by α1, 3/4-focusing transferase (FucT14) derived from *Helicobacter pylori* DMS 6709 (62, 63). A study demonstrated *Bifidobacterium longum* was negatively correlated with LNT by analyzing 170 breast milk HMOs samples and children’s fecal microbiota samples (64). Importantly, we cannot ignore the beneficial effects of these bacteria on the intestinal and immune health of infants. Both *Lactobacillus* and *Bifidobacterium* have the ability to metabolize and break down glycans such as HMOs, which is beneficial for the absorption of HMOs and thereby provides important nutritional support for infants (65). Conversely, HMOs in breast milk can selectively stimulate the proliferation of beneficial bacteria (*Bifidobacterium* and *Lactobacillus*), which is conducive to maintaining intestinal microecological balance and inhibiting the growth of harmful pathogens (66). Therefore, the microbial community together with the HMOs in breast milk constitute a complex, effective defense mechanism that provides the newborn with protection against a variety of potential infections.

The addition of prebiotics, such as 2′-FL, 3′-FL, 3′-SL, and 6′-SL, to infant formula has been approved in the USA (66–69), European Union, and other jurisdictions (70–73). Indeed, HMOs are currently the focus of much research (74–80). Several studies have investigated the safety and tolerability of infant formulas supplemented with HMOs, including those containing one (74, 76), two (77, 78), or five (79, 80) types of HMOs. This study has demonstrated a significant decrease in the total content of HMOs with increasing lactation time, highlighting the importance of colostrum-derived HMOs in breast feeding. We also described the roles of 3′-SL and 6′-SL, which are closely related to breast milk, as well as LNT2. Our findings also provide new evidence and serve as a reference for the addition of certain prebiotic HMO molecules to the development of the next generation of infant milk powders formulated to meet the needs of infants during the transition from breastfeeding to formula.

This study has certain limitations. First, we studied a small number of samples collected at two of the three lactation stages (colostrum and mature milk) from mothers living in only one region. While previous studies investigated the human milk microbiota or HMO content at certain time points during lactation, the focus of this study was the association of the human milk microbiota with the expression of the corresponding HMOs during these two periods. It should also be acknowledged that our analysis did not consider the potential effects of age, lifestyle, maternal diet, presence or absence of the cesarean section, or geographical location on the concentration of HMOs in breast milk (58, 81, 82). Future studies should examine these different parameters on the changes in the human milk microbiota and HMOs during lactation in larger and more diverse cohorts. Additional studies are also needed to understand the associations between the biological functions of specific HMOs and the human milk microbiota over time. Multi-omics analyses (e.g., metabolomics) in combination with culture techniques and multi-site studies, as well as information on infant health outcomes, could be useful to analyze the structure and function of the human milk microbiota in various ways. Further research is urgently needed to elucidate the complex links between the HMO–microbiome and health through microbes, metabolism, and immunity.

In summary, breast milk, which is recognized as a synbiotic, is a highly complex matrix containing a beneficial, dynamic bacterial community and biologically active molecules including HMOs. This study provides new evidence and a fresh perspective on the relationship between breast milk bacteria and HMOs. It is necessary to further explore the mechanisms that drive the establishment and maturation of the intestinal microbiota and the immune system in infants during different lactation periods. Our findings may be useful for the development of powdered infant formula that more accurately mimics the nutritional composition of breast milk.

## Data Availability

The datasets presented in this study can be found in online repositories. The names of the repository/repositories and accession number(s) can be found in the article/Supplementary material.
