# Supplementary material for: Human milk microbiota and oligosaccharides in colostrum and mature milk: comparison and correlation
Source: Front Nutr. 2024 Dec 12;11:1512700. doi: 10.3389/fnut.2024.1512700 (PMC11670000; doi:10.3389/fnut.2024.1512700)
Supplement: Supplementary file 1 [file Table_1.docx]

**Supplementary Table 1 Specific clinical information.**

| ID | Age | Height(cm) | BMI | Weight(kg) | Number of deliveries | Mode of deliveries | Conditions occurring during pregnancy | Dietary information | The application of antibiotics | Infant weight (g) |
| --- | --- | --- | --- | --- | --- | --- | --- | --- | --- | --- |
| C1 | 27 | 160 | 27.34 | 70 | 1 | Cesarean section | Gestational diabetes mellitus | Balanced diet | no | 2850 |
| C2 | 29 | 158 | 24.03 | 60 | 1 | Cesarean section | Diarrhoea | Balanced diet | no | 2950 |
| C3 | 33 | 158 | 29.24 | 73 | 2 | Vaginal birth | Gestational diabetes mellitus | Balanced diet | no | 3400 |
| C4 | 34 | 161 | 33.56 | 87 | 2 | Cesarean section | Normal | Balanced diet | no | 3050 |
| C5 | 32 | 157 | 29.62 | 73 | 2 | Cesarean section | Normal | Balanced diet | no | 3000 |
| C6 | 22 | 158 | 34.45 | 86 | 1 | Cesarean section | Normal | Balanced diet | no | 2950 |
| C7 | 30 | 170 | 20.76 | 60 | 1 | Cesarean section | Normal | Balanced diet | no | 3600 |
| C8 | 35 | 163 | 27.10 | 72 | 2 | Cesarean section | premature birth | Balanced diet | no | 2350 |
| C9 | 33 | 168 | 26.93 | 76 | 1 | Cesarean section | premature birth | Balanced diet | no | 2750 |
| C10 | 27 | 172 | 21.63 | 64 | 1 | Vaginal birth | Gestation-Hypothyroidism | Balanced diet | no | 2400 |
| C11 | 26 | 161 | 27.97 | 72.5 | 1 | Vaginal birth | Normal | Balanced diet | no | 3700 |
| C12 | 35 | 166 | 27.58 | 76 | 1 | Cesarean section | Intrauterine infection | Balanced diet | no | 4000 |
| C13 | 28 | 160 | 26.52 | 67.9 | 2 | Vaginal birth | Gestational diabetes mellitus | Balanced diet | no | 3150 |
| C14 | 27 | 162 | 26.29 | 69 | 1 | Vaginal birth | Normal | Balanced diet | no | 3500 |
| C15 | 39 | 155 | 26.01 | 62.5 | 1 | Cesarean section | Gestation-Hypothyroidism | Balanced diet | no | 3300 |
| C16 | 24 | 172 | 33.40 | 98.8 | 1 | Vaginal birth | Gestation-Hypothyroidism | Balanced diet | no | 3700 |
| C17 | 38 | 169 | 30.32 | 86.6 | 1 | Cesarean section | Prediabetes | Balanced diet | no | 3400 |
| C18 | 41 | 167 | 23.88 | 66.6 | 2 | Cesarean section | Gestational diabetes mellitus | Balanced diet | no | 3550 |
| C19 | 28 | 160 | 24.61 | 63 | 1 | Cesarean section | - | Balanced diet | no | 2900 |
| C20 | 35 | 158 | 30.44 | 76 | 2 | Vaginal birth | Normal | Balanced diet | no | 3200 |
| S1 | 34 | 164 | 22.31 | 60 | 1 | Vaginal birth | Gestational diabetes mellitus | Balanced diet | no | 5000 |
| S2 | 25 | 166 | 30.48 | 84 | 1 | Vaginal birth | Normal | Balanced diet | no | - |
| S3 | 24 | 158 | 23.23 | 58 | 1 | Vaginal birth | Normal | Balanced diet | no | 5900 |
| S4 | 35 | 162 | 28.58 | 75 | 1 | Cesarean section | Normal | Balanced diet | no | 6000 |
| S5 | 28 | 168 | 23.74 | 67 | 1 | Vaginal birth | Gestational diabetes mellitus | Balanced diet | no | - |
| S6 | 25 | 162 | 34.29 | 90 | 1 | Cesarean section | Gestational diabetes mellitus | Balanced diet | no | 4990 |
| S7 | 28 | 163 | 22.58 | 60 | 1 | Cesarean section | Normal | Balanced diet | no | 5300 |
| S8 | 27 | 162 | 25.53 | 67 | 1 | Vaginal birth | Normal | Balanced diet | no | 5500 |
| S9 | 27 | 162 | 21.72 | 57 | 1 | Vaginal birth | Normal | Balanced diet | no | 5100 |
| S10 | 35 | 163 | 24.46 | 65 | 2 | Cesarean section | Gestational diabetes mellitus | Balanced diet | no | 5500 |
| S11 | 38 | 155 | 25.81 | 62 | 1 | Vaginal birth | Normal | Balanced diet | no | 5000 |
| S12 | 36 | 162 | 23.62 | 62 | 2 | Cesarean section | Normal | Balanced diet | no | - |
| S13 | 32 | 158 | 20.03 | 50 | 1 | Cesarean section | Normal | Balanced diet | no | 4900 |
| S14 | 35 | 165 | 23.14 | 63 | 1 | Cesarean section | Normal | Balanced diet | no | 5200 |
| S15 | 31 | 155 | 19.56 | 47 | 1 | Vaginal birth | Normal | Balanced diet | no | 4700 |
| S16 | 32 | 158 | 18.83 | 47 | 1 | Vaginal birth | Normal | Balanced diet | no | 4100 |
| S17 | 35 | 160 | 23.44 | 60 | 1 | Cesarean section | Normal | Balanced diet | no | - |
| S18 | 33 | 168 | 17.72 | 50 | 1 | Cesarean section | Normal | Balanced diet | no | 5500 |
| S19 | 32 | 160 | 17.97 | 46 | 1 | Cesarean section | Normal | Balanced diet | no | 6400 |
| S20 | 30 | 163 | 26.35 | 70 | 1 | Vaginal birth | Normal | Balanced diet | no | - |

**Supplementary Table 2 Statistics of breast milk sequencing samples**

| Sample\Info | Seq_num | Base_num | Mean_length | Min_length | Max_length |
| --- | --- | --- | --- | --- | --- |
| C1 | 58986 | 25296287 | 428.8524 | 249 | 431 |
| C2 | 53643 | 23007391 | 428.8983 | 254 | 431 |
| C3 | 59495 | 25499533 | 428.5996 | 253 | 472 |
| C4 | 57141 | 24505742 | 428.8644 | 244 | 432 |
| C5 | 55158 | 23660998 | 428.9677 | 254 | 435 |
| C6 | 52064 | 22094597 | 424.3738 | 249 | 434 |
| C7 | 62371 | 26587523 | 426.2802 | 254 | 434 |
| C8 | 55405 | 23761557 | 428.8703 | 253 | 432 |
| C9 | 59230 | 25375400 | 428.4214 | 266 | 433 |
| C10 | 64630 | 27329515 | 422.8611 | 250 | 432 |
| C11 | 58839 | 24852324 | 422.3784 | 249 | 431 |
| C12 | 72597 | 31109687 | 428.5258 | 250 | 439 |
| C13 | 58629 | 25150322 | 428.9741 | 404 | 432 |
| C14 | 57128 | 24505052 | 428.9499 | 254 | 434 |
| C15 | 57983 | 24795399 | 427.6322 | 253 | 433 |
| C16 | 56523 | 24249604 | 429.0219 | 254 | 431 |
| C17 | 60504 | 25999494 | 429.7153 | 392 | 434 |
| C18 | 58423 | 24968702 | 427.378 | 253 | 450 |
| C19 | 45577 | 19550736 | 428.9606 | 250 | 432 |
| C20 | 57581 | 24669690 | 428.4346 | 254 | 432 |
| S1 | 53182 | 22589943 | 424.7667 | 225 | 442 |
| S2 | 53939 | 22847358 | 423.5777 | 222 | 432 |
| S3 | 62336 | 26688761 | 428.1436 | 219 | 433 |
| S4 | 74958 | 32160699 | 429.0496 | 222 | 443 |
| S5 | 63876 | 27261580 | 426.7891 | 217 | 435 |
| S6 | 56698 | 23875273 | 421.0955 | 249 | 431 |
| S7 | 72640 | 30881671 | 425.1331 | 248 | 449 |
| S8 | 55529 | 23873881 | 429.9354 | 253 | 436 |
| S9 | 48423 | 20546546 | 424.3138 | 235 | 433 |
| S10 | 84926 | 36140292 | 425.5504 | 249 | 535 |
| S11 | 62569 | 26778190 | 427.9786 | 247 | 514 |
| S12 | 59825 | 25714571 | 429.8299 | 409 | 433 |
| S13 | 73263 | 31252232 | 426.5759 | 225 | 528 |
| S14 | 55841 | 23297127 | 417.2047 | 203 | 501 |
| S15 | 59162 | 25439139 | 429.9912 | 404 | 433 |
| S16 | 87864 | 37621612 | 428.1801 | 249 | 506 |
| S17 | 53741 | 23087494 | 429.6067 | 233 | 434 |
| S18 | 58143 | 24215839 | 416.4876 | 214 | 471 |
| S19 | 56858 | 24357746 | 428.3961 | 200 | 435 |
| S20 | 71149 | 30418208 | 427.5283 | 249 | 431 |

C: the colostrum group (n=20); S: the mature milk (42 days postpartum) group (n=20).

**Supplementary Table 3 Alpha diversity of Breast milk**

| Group | Ace | Chao | Coverage | Shannon | Simpson | Sobs |
| --- | --- | --- | --- | --- | --- | --- |
| C | 63.32±21.78 | 58.84±17.28 | 0.9998±0.0001 | 1.09±0.42 | 0.5433±0.1947 | 52.75±13.87 |
| S | 135.40±108.50^**^ | 132.61±107.84^**^ | 0.9996±0.0005 | 1.41±0.76 | 0.4547±0.2748 | 121.35±98.13^**^ |

^*^*P*<0.05，^**^*P*<0.01，^***^*P*<0.001 compared with the C group.

**Supplementary Table 4 The co-abundance networks on the Genus level**

| name | sg | size | weighted degree | Hub |
| --- | --- | --- | --- | --- |
| g__unclassified_o__Enterobacterales | module_1 | 3.413673 | 2.904612 | 0.000289 |
| g__Elizabethkingia | module_1 | 4.035306 | 4.248302 | 0.004257 |
| g__norank_f__Moraxellaceae | module_1 | 4.083718 | 2.095802 | 0.000054 |
| g__Delftia | module_1 | 4.833281 | 2.07606 | 0.000286 |
| g__Stenotrophomonas | module_1 | 7.32046 | 4.552186 | 0.000321 |
| g__Pseudomonas | module_1 | 9.223246 | 2.11432 | 0.000286 |
| g__Serratia | module_1 | 13.19553 | 4.593043 | 0.000321 |
| g__Gordonia | module_2 | 2.249244 | 0.628889 | 0.000272 |
| g__norank_f__Mitochondria | module_2 | 2.434061 | 2.553987 | 0.024878 |
| g__Paracoccus | module_2 | 2.443666 | 5.128072 | 0.030971 |
| g__unclassified_p__Proteobacteria | module_2 | 3.611988 | 7.358181 | 0.020595 |
| g__Leifsonia | module_2 | 3.973657 | 3.797982 | 0.003624 |
| g__Cutibacterium | module_2 | 4.135011 | 4.558143 | 0.017601 |
| g__Pedobacter | module_2 | 4.844201 | 2.538857 | 0.000781 |
| g__Rothia | module_2 | 7.344853 | 3.892543 | 0.009616 |
| g__Anaerococcus | module_2 | 7.550804 | 5.278783 | 0.017914 |
| g__unclassified_k__norank_d__Bacteria | module_2 | 7.611317 | 5.749994 | 0.005096 |
| g__Corynebacterium | module_2 | 7.883348 | 6.450894 | 0.01853 |
| g__Gemella | module_2 | 9.253511 | 3.257898 | 0.006859 |
| g__Streptococcus | module_2 | 11.0192 | 1.321105 | 0.000961 |
| g__Staphylococcus | module_2 | 11.75208 | 2.727449 | 0.003608 |
| g__Achromobacter | module_2 | 11.82911 | 5.147973 | 0.004661 |
| g__unclassified_f__Alcaligenaceae | module_3 | 0.624326 | 5.296236 | 0 |
| g__Sinomonas | module_3 | 1.478671 | 5.724766 | 0 |
| g__Bradyrhizobium | module_3 | 2.522827 | 5.727534 | 0 |
| g__Sediminibacterium | module_3 | 4.066725 | 6.606697 | 0 |
| g__unclassified_f__Chitinophagaceae | module_3 | 5.731877 | 5.989062 | 0 |
| g__unclassified_f__Comamonadaceae | module_3 | 6.14968 | 2.559864 | 0 |
| g__Sphingomonas | module_3 | 7.498915 | 5.644764 | 0 |
| g__Methylobacterium-Methylorubrum | module_3 | 9.368715 | 6.145495 | 0 |
| g__Undibacterium | module_3 | 11.08579 | 6.243072 | 0 |
| g__norank_f__A4b | module_4 | 0.572736 | 15.92985 | 0.23435 |
| g__Agromyces | module_4 | 0.690363 | 16.60263 | 0.241394 |
| g__Ensifer | module_4 | 0.706411 | 4.163903 | 0.077448 |
| g__Skermanella | module_4 | 0.722282 | 8.760551 | 0.158727 |
| g__Nocardioides | module_4 | 0.73798 | 13.51209 | 0.214496 |
| g__norank_f__norank_o__norank_c__Alphaproteobacteria | module_4 | 0.814007 | 17.19735 | 0.249681 |
| g__Paenisporosarcina | module_4 | 0.828743 | 15.94914 | 0.235609 |
| g__norank_f__JG30-KF-CM45 | module_4 | 0.84333 | 3.349221 | 0.058645 |
| g__Knoellia | module_4 | 0.900247 | 14.32029 | 0.211894 |
| g__norank_f__Vicinamibacteraceae | module_4 | 0.900247 | 7.905117 | 0.127739 |
| g__Brevibacterium | module_4 | 0.900247 | 0.618804 | 0.007455 |
| g__Lysobacter | module_4 | 1.033428 | 14.82673 | 0.224608 |
| g__Porphyrobacter | module_4 | 1.131774 | 20.75969 | 0.274715 |
| g__Sphingobium | module_4 | 1.143609 | 8.380499 | 0.122132 |
| g__Kocuria | module_4 | 1.166991 | 7.944819 | 0.145627 |
| g__Devosia | module_4 | 1.223841 | 12.21077 | 0.189322 |
| g__norank_f__Xanthobacteraceae | module_4 | 1.256906 | 15.42678 | 0.231084 |
| g__Pseudoxanthomonas | module_4 | 1.411736 | 11.02292 | 0.188945 |
| g__norank_f__norank_o__Vicinamibacterales | module_4 | 1.487985 | 12.05128 | 0.19568 |
| g__norank_f__Gemmatimonadaceae | module_4 | 1.577956 | 17.37184 | 0.248653 |
| g__unclassified_f__Rhizobiaceae | module_4 | 1.742636 | 4.563837 | 0.092573 |
| g__norank_f__Blastocatellaceae | module_4 | 2.135319 | 15.2866 | 0.225452 |
| g__Arthrobacter | module_4 | 3.420986 | 9.42096 | 0.162561 |
| g__Brevundimonas | module_5 | 4.894971 | 0.752958 | 0 |
| g__Chryseobacterium | module_5 | 7.524457 | 0.752958 | 0 |
| g__Peptostreptococcus | module_6 | 0.590139 | 3.350984 | 0.029184 |
| g__unclassified_c__Acidobacteriae | module_6 | 0.706411 | 4.339817 | 0.032484 |
| g__Rhodobacter | module_6 | 0.784075 | 1.508801 | 0.002168 |
| g__Exiguobacterium | module_6 | 0.857771 | 5.22094 | 0.052479 |
| g__Novosphingobium | module_6 | 0.872069 | 7.821934 | 0.115356 |
| g__Psychrobacter | module_6 | 0.968374 | 0.601056 | 0.005391 |
| g__Leptotrichia | module_6 | 1.143609 | 7.178495 | 0.104913 |
| g__Alloprevotella | module_6 | 1.478671 | 9.224563 | 0.092366 |
| g__Lawsonella | module_6 | 1.487985 | 6.78768 | 0.077811 |
| g__Fusobacterium | module_6 | 1.629363 | 1.259111 | 0.008628 |
| g__norank_f__Neisseriaceae | module_6 | 1.6461 | 1.566061 | 0.004668 |
| g__Abiotrophia | module_6 | 1.662645 | 3.362105 | 0.030212 |
| g__Nitrospira | module_6 | 1.897436 | 7.615881 | 0.092987 |
| g__Actinomyces | module_6 | 3.466455 | 4.523448 | 0.056474 |
| g__Porphyromonas | module_6 | 3.662217 | 8.569777 | 0.111229 |
| g__Veillonella | module_6 | 4.17015 | 1.257219 | 0.015298 |
| g__Peptoniphilus | module_6 | 4.200211 | 15.82302 | 0.157214 |
| g__Prevotella | module_6 | 4.44222 | 15.70136 | 0.220053 |
| g__norank_f__norank_o__Chloroplast | module_6 | 4.482383 | 0.628234 | 0.003296 |
| g__Enhydrobacter | module_6 | 5.369504 | 0.615275 | 0.012842 |
| g__Granulicatella | module_6 | 5.581746 | 4.831192 | 0.034327 |
| g__Neisseria | module_6 | 5.937485 | 9.9695 | 0.07144 |
| g__Finegoldia | module_6 | 6.996511 | 0.617863 | 0.001763 |
| g__Thermus | module_6 | 7.27562 | 1.40726 | 0.007323 |
| g__Haemophilus | module_6 | 7.737964 | 5.983507 | 0.046992 |
| g__Thermomonas | module_7 | 0.624326 | 1.329819 | 0 |
| g__TM7a | module_7 | 0.857771 | 0.688359 | 0 |
| g__unclassified_f__Dermacoccaceae | module_7 | 1.440804 | 0.64146 | 0 |
