# Supplementary material for: Human milk microbiota and oligosaccharides in colostrum and mature milk: comparison and correlation
Source: Front Nutr. 2024 Dec 12;11:1512700. doi: 10.3389/fnut.2024.1512700 (PMC11670000; doi:10.3389/fnut.2024.1512700)
Supplement: Supplementary file 2 [file Image_1.pdf]

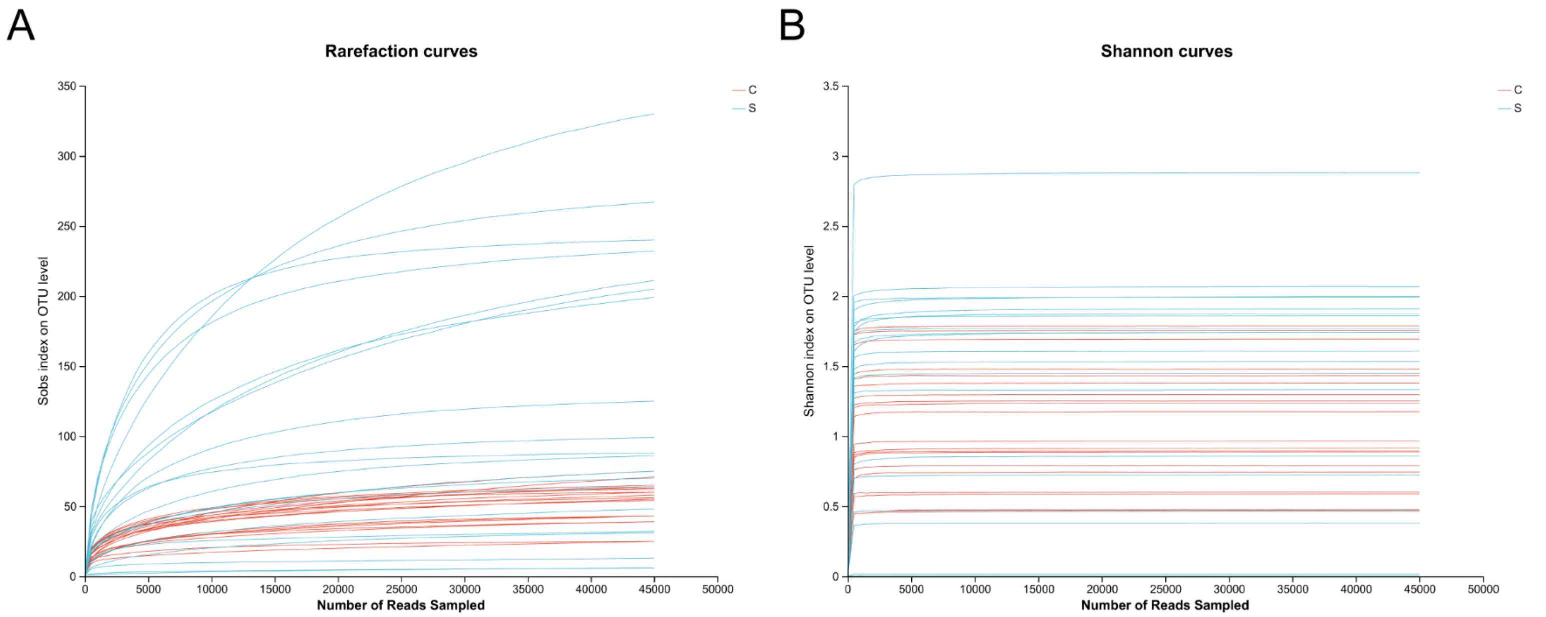

Supplementary Figure 1 Refraction curve of the (A) the Sobs index and (B) the Shannon index. C: the colostrum group (n=20); S: the mature milk (42 days postpartum) group (n=20).

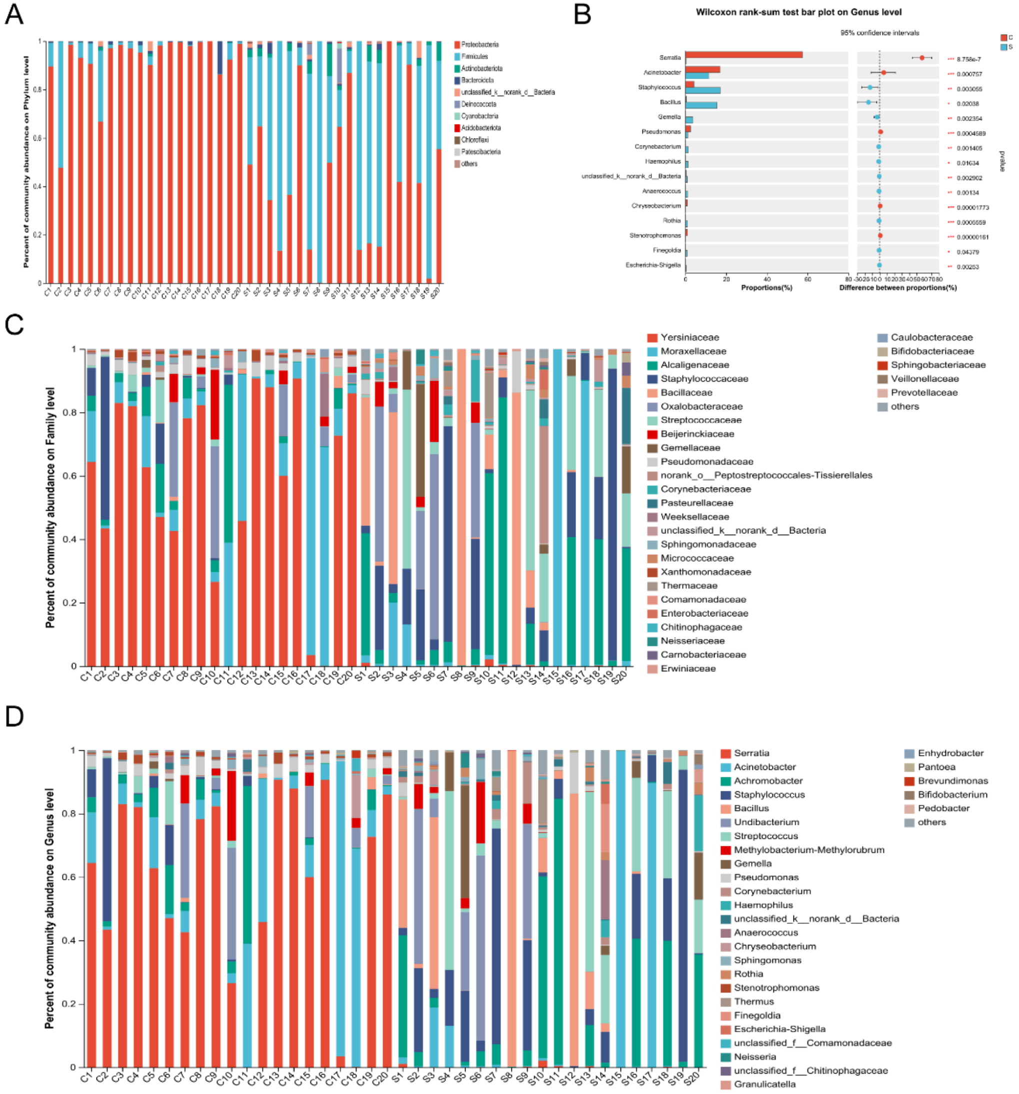
A
Percent of community abundance on Phylum level
C1 C2 C3 C4 C5 C6 C7 C8 C9 C10 C11 C12 C13 C14 C15 C16 C17 C18 C19 C20 S1 S2 S3 S4 S5 S6 S7 S8 S9 S10 S11 S12 S13 S14 S15 S16 S17 S18 S19 S20
Proteobacteria
Firmicutes
Actinobacteriota
Bacteroidota
unclassified_k__norank_d__Bacteria
Deinococcota
Cyanobacteria
Acidobacteriota
Chloroflexi
Patescibacteria
others
B
Wilcoxon rank-sum test bar plot on Genus level
95% confidence intervals
C
S
Serratia *** 8.758e-7
Acinetobacter *** 0.000757
Staphylococcus ** 0.003055
Bacillus * 0.02038
Gemella ** 0.002354
Pseudomonas *** 0.0004589
Corynebacterium ** 0.001405
Haemophilus * 0.01634
unclassified_k__norank_d__Bacteria ** 0.002902
Anaerococcus ** 0.00134
Chryseobacterium *** 0.00001773
Rothia *** 0.0005559
Stenotrophomonas *** 0.00000161
Finegoldia * 0.04379
Escherichia-Shigella ** 0.00253
Proportions(%)
Difference between proportions(%)
pvalue
C
Percent of community abundance on Family level
Yersiniaceae
Moraxellaceae
Alcaligenaceae
Staphylococcaceae
Bacillaceae
Oxalobacteraceae
Streptococcaceae
Beijerinckiaceae
Gemellaceae
Pseudomonadaceae
norank_o__Peptostreptococcales-Tissierellales
Corynebacteriaceae
Pasteurellaceae
Weeksellaceae
unclassified_k__norank_d__Bacteria
Sphingomonadaceae
Micrococcaceae
Xanthomonadaceae
Thermaceae
Comamonadaceae
Enterobacteriaceae
Chitinophagaceae
Neisseriaceae
Carnobacteriaceae
Erwiniaceae
Caulobacteraceae
Bifidobacteriaceae
Sphingobacteriaceae
Veillonellaceae
Prevotellaceae
others
D
Percent of community abundance on Genus level
Serratia
Acinetobacter
Achromobacter
Staphylococcus
Bacillus
Undibacterium
Streptococcus
Methylobacterium-Methylorubrum
Gemella
Pseudomonas
Corynebacterium
Haemophilus
unclassified_k__norank_d__Bacteria
Anaerococcus
Chryseobacterium
Sphingomonas
Rothia
Stenotrophomonas
Thermus
Finegoldia
Escherichia-Shigella
unclassified_f__Comamonadaceae
Neisseria
unclassified_f__Chitinophagaceae
Granulicatella
Enhydrobacter
Pantoea
Brevundimonas
Bifidobacterium
Pedobacter
others

Supplementary Figure 2 Analysis of the composition of breast milk microbiota. (A) Microbial distributions of each sample at the phylum level. (B) Species difference analysis using the Wilcoxon rank-sum test at the genus level. (C) Microbial distributions of each sample at the family level. (D) Microbial distributions of each sample at the genus level. C: the colostrum group (n=20); S: the mature milk (42 days postpartum) group (n=20). $^{*}P$<0.05， $^{**}P$<0.01， $^{***}P$<0.001 compared with the C group.

Spearman Correlation Heatmap

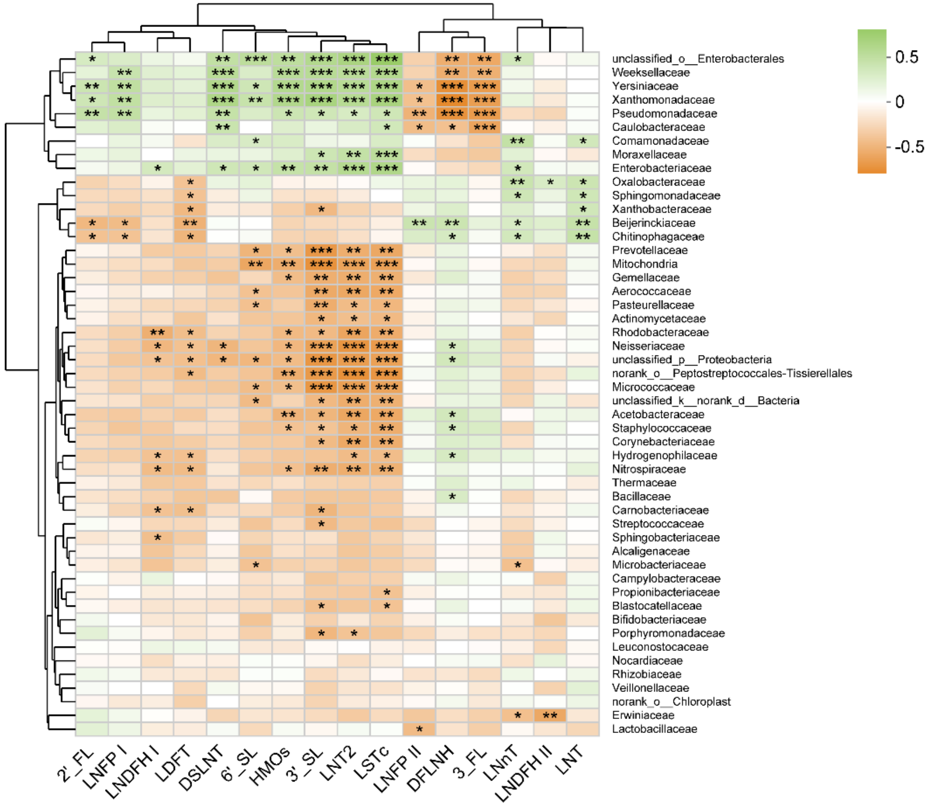

Supplementary Figure 3 Analysis of the correlation between oligosaccharides and the breast milk microbiota at the family level. C: the colostrum group (n=20); S: the mature milk (42 days postpartum) group (n=20). $^{*}P<0.05$， $^{**}P<0.01$， $^{***}P<0.001$.
